# Is Distributional Justice Equivalent to Prosocial Sharing in Children’s Cognition?

**DOI:** 10.3389/fpsyg.2022.888028

**Published:** 2022-07-12

**Authors:** Yuning Zhu, Jingmiao Zhang, Xiuli Liu

**Affiliations:** ^1^School of Psychology, Northeast Normal University, Changchun, China; ^2^School of Educational Science and Technology, Anshan Normal University, Anshan, China

**Keywords:** distribution, sharing, confusion, distinguish, connection

## Abstract

Distribution and sharing are social preference behaviors supported and shaped by selection pressures, which express individuals’ concern for the welfare of others. Distributive behavior results in distributive justice, which is at the core of moral justice. Sharing is a feature of the prosocial realm. The connotations of distribution and sharing are different, so the principles, research paradigms, and social functions of the two are also different. Three potential causes of confusion between the two in the current research on distribution and sharing are discussed. First, they share common factors in terms of individual cognition, situation, and social factors. Second, although they are conceptually different, prosocial sharing and distribution fairness sensitivity are mutually predictive in individual infants. Similarly, neural differences in preschoolers’ perception of distribution fairness predict their subsequent sharing generosity. Finally, similar activation regions are relevant to distribution and sharing situations that need behavioral control on a neural basis.

## Introduction

The development of children’s resource distribution and sharing behavior has been widely concerned by researchers of developmental psychology. Distribution and sharing are social preference behaviors supported and shaped by selection pressures (Silk and House, 2016), expressing individuals’ concern for the welfare of others. Across a series of studies, the violation-of-expectation paradigm was used to investigate the detection of distribution norm violation in infants (Geraci and Surian, 2011; Meristo and Surian, 2013; Burns and Sommerville, 2014; Buyukozer et al., 2019). Infants at 4, 9, and 10 months of age looked at the unequal 2:0 distributions significantly longer than the equal 1:1 distributions (Meristo and Surian, 2013). However, they looked at no significant difference between the 3:1 and 2:2 distributions (Buyukozer et al., 2019). Infants’ increased sensitivity to distributive justice occurs between 6 and 12 months (Ziv and Sommerville, 2017). The 15-month-old infants looked the unfair outcome (the 3:1 distribution) significantly longer than the fair outcome (the 2:2 distribution), indicating increased sensitivity to the distributive fairness of the third parties (Burns and Sommerville, 2014).

At present, there are few studies on prosocial sharing behavior in infants. It may due to the limited social development level of infants, the naturalistic sharing rarely arises in infants. According to parents’ reports in interviews, naturalistic sharing occurs as early as 9 months of age (Ziv and Sommerville, 2017). In the prompted giving task, infants at 15 and 18 months of age were able to engage in sharing behavior after being presented with a series of progressively more explicit cues (Schmidt and Sommerville, 2011; Brownell et al., 2013). The higher the sensitivity of 15-month-old infants to the fairness of third-party distribution, the more inclined they are to perform altruistic sharing (sharing their favorite toys). In contrast, infants with less sensitivity tend to share selfishly in the prompted giving task (sharing toys they do not like; Schmidt and Sommerville, 2011). Moreover, the change in infants’ concern about distribution equity can be predicted by their tendency to generously share their favorite toys (Sommerville et al., 2013; Ziv and Sommerville, 2017).

According to the developmental characteristics of children’s ownership understanding, children before the age of 4 have limited understanding and reasoning ability of ownership. If the ownership of resources in experimental situations is not clear, children will think that the resources in different situations belong to themselves, so there is no difference in the number of resources distributed to others in different situations (Hamann et al., 2011; Wu et al., 2017). With the increase of age, the individual’s ability of ownership understanding gradually improves. When children own resource ownership or resource ownership is not unique to children, the behavior of the two situations gradually appears different. That is, when the ability of ownership understanding is mature, according to the ownership of resources, when resources are jointly owned, children’s behavior of distributing resources to others needs to consider fair distribution; When the ownership of resources belongs to themselves, the behavior of children sharing resources to others is a kind of prosocial altruistic sharing, which gradually develops into two different social behaviors. The former is distribution, involving fairness and justice, belonging to the field of morality; The latter is sharing, which is an altruistic behavior.

## There Is Confusion Between Distribution and Sharing in Research

Although distribution and sharing have different meanings and belong to different fields, In developmental psychology, there is confusion regarding distribution and sharing in children.

Our analysis of the literature led us to identify four types of confusion between distribution and sharing. The first type of confusion occurs when researchers confuse the two concepts. For example, prosocial sharing situations appear in studies of the characteristics of children’s distributive justice behavior. For instance, experimenters may explicitly instruct the child that the resources belong to him/her, and he/she could choose to share or not to share them with others (Blake and Rand, 2010; Wang and Su, 2013; Li et al., 2014; Reis and Sampaio, 2019; Urbanska et al., 2019). The behavior thus produced would be sharing behavior, in the prosocial domain. Then too, in study of children’s sharing behavior, researchers use an allocation design (Hamann et al., 2011; Steinbeis, 2016; Yu et al., 2016; Ji and Gao, 2017; Vonk et al., 2020). In an experimental instruction, if a child is informed that the resources belong to him/her and another recipient or the ownership of the resources are not explained but only that it is up to him/her how the resources are divided, changes the setup of the experiment. That is to say, the setting in which the behaviors occur is irrelevant, and the resulting behaviors may not be what the researchers hope to observe.

In the second type, in study of distribution or sharing behavior, some researchers are unaware of potential confusion in distribution or sharing behavior, which tends to lead researchers to take the sharing research results as the evidence for the development of distribution behavior in the context of children’s idea of distributive justice (Paulus and Essler, 2020). By the same token, when discussing the developmental characteristics or neural basis of children’s prosocial sharing behavior, findings regarding distributive justice are taken as evidence of prosocial sharing behaviors (Blake, 2018; Steinbeis, 2018; Meng and Moriguchi, 2021).

The third type of confusion is that in the study of distribution justice, researchers have insufficiently analyzed and discussed their results in relation to moral justice but regard distributive justice as a prosocial behavior (Kanngiesser and Warneken, 2012; Smith et al., 2013). However, distribution is an economic term, and distributive justice is in a moral category, so it is inappropriate to discuss the results from a prosocial viewpoint.

The last confusion relates to the fact that the forms of distribution and sharing in dictator’s game scenarios are similar, although they are essentially different. Some researchers have unified the concepts of distribution and sharing in their research, dividing prosocial sharing into the categories of autonomous sharing and obligation/responsibility sharing (Wu et al., 2017). Here, autonomous sharing means that children share their resources, as these come to the recipient thanks to a personal effort. Moreover, obligation/responsibility sharing describes a cooperative situation in which children have the obligation or responsibility to distribute resources that come from the joint effort of both parties. Because the joint efforts of both parties obtain the resources, this is a distributive situation, and it is only appropriate for studying distributive behavior.

From sorting, analyzing, and summarizing the relevant core literature, we put forward a few likely causes of the above confusions. First, distribution and sharing may have common influencing factors, divided into three aspects: individual, situational, and social. These factors can affect both distribution and sharing. Second, an internal relationship appears between distribution and sharing. It has been found that whether and how early individuals share can predict their sensitivity to distributive fairness. Third, both sets of behaviors may have a similar neural basis.

## Differences Between Distribution and Sharing

### The Concept

Distribution refers to the division of social resources, wealth, responsibilities, and obligations in social groups according to certain standards or regulations. It is a process of allocation of social and economic resources. In developmental psychology, the study of distribution among children is mainly concerned with the idea of distributive justice, that is, children’s understanding and application of the criteria to be used to distribute resources (Hsu et al., 2008). The premise of distribution is that the allocated resources are owned by the society or the collective, not by individuals. Therefore, individual distribution is a decision-making behavior that considers the interests of the self and others at the same time (Dijk and Vermunt, 2000; Leliveld et al., 2008; Chernyak et al., 2019).

Sharing refers to both having and using things owned by individuals, whether goods or more abstract entities, like rights, emotions, and experiences, with others (Chen et al., 2004; Liao, 2014). In developmental psychology, research on children’s sharing focuses on children’s willingness and behavior to give part of their possessions to others. Sharing behavior, which is an important part of prosocial behavior, is an important indicator of individual socialization.

### The Principle

In distribution, the resources allocated usually do not belong to the distributor but to the collective or society. Following different social goals, the distribution follows different principles, among which the most prominent are the three principles of equality, equity and need (Deutsch, 1975). The ultimate fairness of the distribution involves moral evaluation. We must consider the issue of distributive fairness, that is, distributive justice. The concept of distributive justice relates to the distribution of social benefit and social obligations. Distribution must not be arbitrary, and the corresponding distribution principles must be followed to achieve fairness in the distribution results or procedures. A distributor must abide by such principles in the distribution, or a recipient or a third party may exact punishment.

Sharing behavior is more common in daily life, and it has no specific criterion or principle. Generally, individuals consider themselves to have the right to decide whether and how much of their own to give to others, holding that neither society at large nor individual others have the right to control whether and how they share. Furthermore, no punishment is indicated for the sharer no matter how many resources the recipient receives.

### Research Paradigm

Psychological research on children’s distribution behaviors mainly uses an economic game paradigm, namely, the dictator game, ultimatum game, and third-party tasks. The self-interest of the individual is activated in the first two contexts. In the third-party task, the subject observes resource allocation performed by others or allocates resources to others, in a context without self-interest at play. In distribution as an economic concept, its related behavior consequences involve distributive justice, so distributive behavior involves moral evaluation (McAuliffe et al., 2017).

In the study of sharing behavior, because the sharer owns the resource, all of the specific aspects of sharing involve the individual’s interests. Thus, researchers generally adopt a variation on the dictator game. In a sharing situation, children are allowed to share their items with others unconstrainedly, and this can be used to study individual prosocial motivation and behavior (Benenson et al., 2007). Earlier studies of children’s sharing behavior have gathered data from natural observation, teacher or parent reports, and interviews.

### Social Function

Because distribution and sharing belong to different fields, their social functions may be disparate. The equitable allocation of resources is conducive to maintaining social order, promoting cooperation and the development of civilization (Baumard et al., 2013; Decety and Yoder, 2017). Prosocial behavior can help individuals build good social relationships, eliminate individual negative emotions, cope with psychological stress, and improve well-being. The concept of the warm glow proposed by Andreoni refers to the good feeling, satisfaction, or happiness generated by prosocial behavior. This effect can explain the difference between altruistic and non-altruistic behavior in an individual. Cross-cultural and child development research strongly supports a universal relationship between prosocial behavior and well-being (Aknin et al., 2013). Studies in children have shown that sharing can positively impact mood and that sharing behavior activates brain regions associated with reward (Cutler and Campbell-Meiklejohn, 2019). Before children engage in sharing behavior, they expect that it will produce positive emotions; further, autonomous sharing can improve children’s subjective well-being (Aknin et al., 2012; Wu et al., 2017; Sabato and Kogut, 2019). These findings suggest that individual well-being increases after prosocial behavior, which may be a common proximal mechanism for such behavior and might provide a theoretical explanation for the emergence of early prosocial behavior (Paulus and Moore, 2017).

Some researchers have suggested that there may be a positive feedback loop between positive emotions and prosocial behavior. A previous study that examined the relationship between positive emotions and donating behavior in children aged 7–8 years found that children who imagined happy events donated significantly more than children who imagined sad ones or control groups. (Moore et al., 1973). A study that used short video clips to induce sadness in children aged 5–6 years found that sadness significantly reduced boys’ sharing behavior, but it did not affect girls’ sharing behavior (Guo et al., 2019). Past studies have shown that positive states predict prosocial behavior and prosocial behavior predicts positive states, but few studies have investigated these relationships together in one experiment (Aknin et al., 2018).

Research on the effects of distributive behavior on children’s positive emotions is still lacking. Wu et al. (2017) found that obligation sharing does not affect an individual’s positive emotions, that is, it cannot improve the individual’s subjective well-being, while autonomous sharing can. The resources at play in obligation sharing situation are obtained through the cooperation of two children, meaning that this is actually a case of distribution. That is, it is different from prosocial sharing. Whether the relationship between distribution and positive emotion is similar to the relationship between sharing and positive emotion should be explored in future work.

## Connection Between Distribution and Sharing

### Common Influencing Factors

#### Individual Cognitive Factors

##### Theory of Mind

Theory of mind (TOM) is an important cognitive factor of children’s distributive justice and prosocial sharing. The multiple forces hypothesis indicates that TOM can help children balance their self-interest and the needs of others in distributive situations (Chen and Wu, 2017). Children who have passed the second-order false belief task allocated more resources to strangers than those who have not. Second-order false beliefs, likewise, did not affect children’s allocation to friends and relatives (Yu et al., 2016). These findings are consistent with the multiple forces hypothesis. However, the TOM predicted that children would distribute more stickers to their friends over time (Vonk et al., 2020). Priming children’s speculation on recipients’ goals in the competitive situation could significantly affect children’s allocation behavior and reduce children’s resources allocated to competitors (Nilsen and Valcke, 2018). Inducing children’s perspective taking significantly increases the rejection of unfair distribution (Tsoi and McAuliffe, 2019).

The multiple forces hypothesis indicates that the effect of the TOM is not obvious in relatively simple sharing situations. A meta-analysis found that children’s TOM was significantly associated with helping and cooperative among prosocial behaviors but not with sharing behaviors (Imuta et al., 2016). Nevertheless, in more complex sharing situations, TOM allows children to understand each other’s needs more accurately and quickly, thus promoting prosocial sharing behavior (Kogut et al., 2016). These results may indicate that when children have clear social norms to follow, the relationship between TOM and sharing behavior is no longer significant.

#### Situational Factors

The possible common situational factors for distribution and sharing mainly include the means of resources acquisition and social reputation.

##### Means of Resources Acquisition

There are two main ways in which for children can obtain resources in an allocation situation: windfall gains and earned rewards. The former are directly provided by adults, making it a windfall for the distributor and recipient. The latter are obtained through effort (participation in collaborative or parallel work). In the case of windfall, infants in the first year of life already have the sensitivity to the fairness of third-party distribution (Buyukozer et al., 2019). The infants expect a reward to be distributed by a third party to the person who protects the victim from attack (Geraci, 2020; Geraci and Surian, 2021). Similarly, infants expect third parties to allocate comforter rewards, that go beyond the principle of equality (Geraci et al., 2021). Studies the use provided by adults have shown that disadvantageous inequity aversion appears around 4 years old, and advantageous inequity aversion appears at 8 years old (Blake and McAuliffe, 2011; McAuliffe et al., 2013). When the resources were obtained by the children’s own efforts, however, they can spontaneously distributed the rewards of cooperation equally among everyone as early as 3 years old. When they worked in parallel and received their rewards separately, they accepted the inequality of the results (Hamann et al., 2011). In short, children’s distributive equity is particularly sensitive to means of resource acquisition.

In a prosocial sharing study in children, researchers divided the resources into things occasionally gained and things one possesses according to the way in which they were acquired. The things occasionally gained were directly provided by the experimenter and thus were windfalls for the sharer; things one possess marked the rewards that children earned through their own hard work. The probability of natural sharing is very low in infancy regardless of the item. According to parents’ reports in interviews, naturalistic sharing occurs at 9 months of age (Ziv and Sommerville, 2017). In the laboratory, infants at 15 and 18 months of age were able to engage in sharing behavior after being presented with a series of progressively more explicit cues (Schmidt and Sommerville, 2011; Brownell et al., 2013; Ziv and Sommerville, 2017). Studies found that preschool children are more willing to share things that are occasionally gained (Wang et al., 2005; Liu et al., 2013). Another study found that preschoolers shared prizes they earned through hard work the most, followed by their favorite toys, and least of all occasionally gained food (Li and Zhao, 2008). This study may be inconsistent with others may be that everything occasionally gained is plasticine, and the items accidentally obtained are small pieces of food. It is possible that different types of resources may entail different levels of attraction to children. For another, it may be that toys are not shared in the same way as food. Therefore, the resource types should be unified in future sharing studies.

##### Social Reputation

Social reputation relates to how far children are willing to make a reasonable allocation of resources in the name of equity and represent prosocial sharing that meets social expectations. In the distribution situation, children’s social reputation indicates that when children realize that others may judge their distribution behavior, they adjust this behavior to behave more fairly to obtain a positive evaluation from others.

Many studies have shown that 5-year-old children are more generous in the presence of peers than when no one is present. When they are with different people, they make different allocation decisions (Dunham et al., 2011; Engelmann et al., 2012; Leimgruber et al., 2012). Children aged 6–8 years may be more concerned with their social reputation, making it more likely that they will behave fairly when their peers are present or when experimenters can learn of their choices. However, if the distribution that they give will not be found by others, the originally fair children may become unfair. In other words, the fair distribution is partly due to the children’s desire to appear fair in front of others (Shaw et al., 2014). Children aged 6–9 are more likely to accept an advantageous distribution if their peers are unaware of their advantages (McAuliffe et al., 2020). The results of these studies show that even if children have learned fair norms, internalized norms may also be strategically used in social situations that can improve their reputation. Social reputation concerns can narrow the knowledge and behavior gap in fair distribution among children.

Children’s concern for their social reputation can affect their sharing behavior in different sharing situations. In prosocial research, an individual’s reputation refers to others’ evaluation of his or her prosocial ability and motivation, such as whether others consider that he or she often treats others generously (Engelmann and Rapp, 2018). Children share more resources when they are aware of the presence of the recipient or other observers than when they are not; that is, children are more generous when they realize that their behavior is observed by others (Leimgruber et al., 2012; Sampaio and Neto Pires, 2015). In addition, because 5-year-olds are concerned about their reputation within the in-group, they tend to share more resources when observed by members of their in-group (Engelmann et al., 2013). More recently, study found that 5- to 9-year-old children’s sharing with in- and out-group members was affected by reputation in all groups (Yazdi et al., 2020). In conclusion, children’s attention to their reputation can affect their generosity in the context of prosocial sharing.

#### Social Factors

The social factors that could affect distribution and sharing mainly include social distance and social culture.

##### Social Distance

Social distance reflects the level and degree of closeness or alienation between people and groups. In one study, children aged 3–6 gave significantly more stickers or candy to their friends than to unfamiliar children whom they have never met (Yu et al., 2016). In the event of conflict between social relationships and contributions to allocation, children younger than seven decide based on social relationship. They adjust these allocation decisions in relation to the size of the recipient’s contribution (Zhang, 2020). At the group level, social distance affects individuals’ response to the unfair behavior of in- and out-group members, and individuals show in-group preferences even if in-group members violate the distribution principle (Zhang and Zhao, 2018). Blake generally explains these findings as indicating a social distance effect, which means that individuals can give more resources to recipients who are more closely related to them (Charness and Gneezy, 2008; Wu et al., 2011; Blake, 2018).

Children will allocate different amounts of resources to different recipients in the process of sharing according to the closeness of the relationship with the recipients. Preschool children aged 3–6 years share more rewards with their friends than non-friends and strangers (Vonk et al., 2020). In another study, in the exploration of whether preschool children choose to share with friends out of reciprocity, the researchers found that there were no differences between 3- and 5-year-olds in giving to friends with and without reciprocity, which shows that children’s preference to share with friends is independent of reciprocity (Lenz and Paulus, 2021). More studies are needed to explore the specific effects of social distance on children’s prosocial sharing.

##### Social Cultural

The development of individual social behavior cannot be separated from the social cultural environment. Cross-cultural study of distribution indicates that advantageous inequity aversion varies from culture to culture. In some cultures, advantageous inequity aversion appears in middle childhood, but it not in others (Blake et al., 2015). When a quantity of items presented cannot be distributed equally, children would rather throw away some items than distribute them unfairly. However, unlike American children, Ugandan children tend to allocate resources unfairly rather than throw away extra resources (Paulus, 2015). Moreover, in Uganda, preschool and primary school children show a high level of generosity independent of social relationships (Scharpf et al., 2016). When recipients are different in wealth and contribution, children from individualistic cultures are more likely than those from collectivist cultures to favor fair distribution (more to the poor and to those with greater merit) than equal distribution. When recipients differ in degrees of injury, children from more collectivist culture tend to allocate more resources to more injured recipients than children from more individualistic cultures (Huppert et al., 2019). Recent studies have found that the role of merit in distribution seems to be different across cultures. Compared with Kenyan children, Chinese and German children selectively allocate resources to individuals who have more work. When friendship and merit are opposed, in all three cultures, children tend to share equally between friends who contribute less and less familiar people who contribute more (Engelmann et al., 2021). These results indicated that both commonality and individuality factor into individual equity in different cultures, which illustrates the significance of cross-cultural research in understanding the development of human distribution equity.

There have been few studies on the impact of social culture on sharing. Previous studies have shown that Asian children are more likely to share spontaneously and less likely to share passively (Rao and Stewart, 1999). Children in collectivist cultures tend to live in communities where harmonious interaction is highly valued, and they are more likely to share with their peers than children in individualistic cultures are (Stewart and McBride-Chang, 2000). However, a study on the sharing behavior of nearly 2,500 children aged 3–12 years from 12 countries on five continents did not find the expected significant differences between children from collectivist and from individualist countries (Samek et al., 2020). Therefore, more research is needed to determine whether social culture has an impact on children’s prosocial sharing behavior and internal mechanisms.

### Possible Mutual Prediction

Infant studies have found that distribution and sharing can predict each other. The higher the sensitivity of 15-month-old infants to the fairness of third-party distribution, the more inclined they are to perform altruistic sharing (sharing their favorite toys). In contrast, infants with less sensitivity tend to share selfishly in the prompted giving task (sharing toys they do not like; Schmidt and Sommerville, 2011). Moreover, the change in infants’ concern about distribution equity can be predicted by their tendency to generously share their favorite toys (Sommerville et al., 2013; Ziv and Sommerville, 2017). What is more, the relationship between infants’ sharing behavior and their sensitivity to distributive justice cannot be predicted by developmental maturity or their cognitive performance (receptive vocabulary; Sommerville and Enright, 2018). To explain the connection between distribution and sharing in infants, the researchers believe that individual sharing interactions provide rich learning opportunities for studying the core principle of distribution. In the interactions they have the opportunity to experience being either the subject or recipient of fair and unfair behavior. These experiences can help them understand the consequences of inequity, making them pay closer attention to the results of unfair distribution (Sommerville and Enright, 2018).

The relationship between distribution and sharing indicates an important aspect of the relationship between the moral and prosocial fields. It has been found that the late positive potential, more than the early posterior negativity, of moral situation processing can predict the actual generosity of children’s later sharing, while children’s moral evaluation can predict their generosity of sharing (Cowell and Decety, 2015). The neural difference in distribution fairness and unfairness in early adolescence can predict children’s participation in subsequent donation behavior, such that the greater the neural difference between them, the longer that children will persist in participating in donation behavior (Meidenbauer et al., 2018). This is consistent with previous findings that indicate that children’s moral reasoning is related to prosocial sharing (Stewart and McBride-Chang, 2000). In conclusion, children’s moral development is closely related to prosocial development, so there is sufficient reason to speculate that there may be a close internal relationship between children’s distribution and sharing behaviors.

### Similar Neural Basis

In distribution, individuals solve conflicts between self-interest and fairness by following social norms, which require ability behavioral control. Neuroscientific studies have shown that increased individual norm compliance is strongly positively correlated with the activation of the lateral orbitofrontal cortex and the right dorsolateral prefrontal cortex (rDLPFC; Spitzer et al., 2007). Disrupting the right DLPFC, but not the left, by non-invasive low-frequency repetitive transcranial magnetic stimulation significantly reduces subjects’ willingness to refuse unfair propose without affecting their perception of fairness (Knoch et al., 2006; Ruff et al., 2013). Longitudinal structural magnetic resonance imaging (sMRI) studies have shown that DLPFC takes a long time to mature, completing its development in early adulthood (Gogtay et al., 2004; Shaw et al., 2008). Therefore, the researchers believe that young children’s violation of the principle of distributive justice is not due to a lack of understanding of right and wrong but rather to the inability to implement behavior control when tempted by resources. The implementation of self-control depends on the function of the mature brain regions in late ontogeny (Steinbeis et al., 2012). With the maturity of individual brain development, individual self-control ability is gradually enhanced. Therefore, the distribution principle is followed in a broad context, and eventually, the principle will apply to others and to the children themselves (McAuliffe et al., 2017).

There is also a conflict between one’s own interests and those of others in sharing situations. However, there have been few studies on the neural basis of sharing behavior. A recent study used functional near-infrared spectroscopy (fNIRS) to record the activation of the DLPFC during sharing in children, which found that DLPFC was activated during cognitive tasks involving behavioral control and sharing tasks involving equal rather than more selfish sharing (Meng and Moriguchi, 2021). This suggests that generous sharing requires self-control, and children’s cerebral cortex is activated in a similar way to the case of the distribution situation. Hence, more research is needed in future to explore the neural basis of sharing.

## Conclusion

Taken together, this review shows that distribution and prosocial sharing differ in their connotation, principles, and social function, but both involve trade-offs between one’s own and others’ interests. Considerable research has been done on the development of resource allocation behavior in children, but we need to conduct more prosocial sharing research to explore the early developmental origins of both. In recent years, studies have been conducted to compare the two sets of behaviors in terms of motivation and emotion (Krettenauer et al., 2019) and explore the relationship between distributive justice and generous sharing from moral and prosocial perspectives (Meidenbauer et al., 2018), which are the main avenues for future research.

## Author Contributions

YZ, JZ, and XL contributed to conception and design of the review. YZ and JZ wrote sections of the manuscript. All authors contributed to the article and approved the submitted version.

## Funding

Preparation of this paper was supported by the Children’s Empathy Development Program (grant no.130021820) and the Children’s Empathy Ability Program (grant no.130013920) of Northeast Normal University.

## Conflict of Interest

The authors declare that the research was conducted in the absence of any commercial or financial relationships that could be construed as a potential conflict of interest.

## Publisher’s Note

All claims expressed in this article are solely those of the authors and do not necessarily represent those of their affiliated organizations, or those of the publisher, the editors and the reviewers. Any product that may be evaluated in this article, or claim that may be made by its manufacturer, is not guaranteed or endorsed by the publisher.
